# One-hour positive pressure ventilation after a successful spontaneous breathing trial: a multicenter feasibility randomized clinical trial

**DOI:** 10.62675/2965-2774.20250361

**Published:** 2025-05-04

**Authors:** Aline Braz Pereira, Michelli Marcela Dadam, Bruna de Albuquerque Catelano, Daniela Delvan, Vitor Hugo Silva Pastorello, Luana Caroline Radun, Israel Silva Maia, Cassio Luis Zandonai, Eliana Vieira Santucci, Gabriela Souza Murizine, Marcelo Luiz Pereira Romano, Glauco Adrieno Westphal, Alexandre Biasi Cavalcanti

**Affiliations:** 1 Centro Hospitalar Unimed Intensive Care Unit Joinville SC Brazil Intensive Care Unit, Centro Hospitalar Unimed - Joinville (SC), Brazil.; 2 Hospital Municipal São José Intensive Care Unit Joinville SC Brazil Intensive Care Unit, Hospital Municipal São José - Joinville (SC), Brazil.; 3 Hospital Nereu Ramos Intensive Care Unit Florianópolis SC Brazil Intensive Care Unit, Hospital Nereu Ramos - Florianópolis (SC), Brazil.; 4 HCor-Hospital do Coração Research Institute São Paulo SP Brazil Research Institute, HCor-Hospital do Coração - São Paulo (SP), Brazil.

## INTRODUCTION

A small, randomized trial suggests that 1-hour positive pressure ventilation after a successful spontaneous breathing trial (SBT) on a T-piece may decrease extubation failure.^( 1 )^ However, the effect on extubation failure was not statistically significant in another similar trial, despite a possible benefit for the subgroup of patients with more than 72 hours of mechanical ventilation (MV).^( 2 )^ The primary objective of this study was to evaluate the feasibility of conducting large randomized controlled trials to determine whether, in patients with more than 72 hours of MV, 1-hour positive pressure ventilation following a successful SBT, either on T-piece or pressure support, reduces the risk of extubation failure within 7 days. Feasibility was defined as the capability to complete the study according to the planned schedule (enrolment within 6 months) and with adherence above 90% to the procedures in the experimental group (1-hour positive pressure ventilation [± 10 minutes] after an SBT followed by extubation) and control group (immediate extubation after an SBT).

## METHODS

This randomized, multicenter feasibility (pilot) trial was conducted at four Brazilian hospitals. Patients and healthcare professionals were not blinded to treatment assignments. The trial was approved by the Ethics Committee of the coordinating center and the sites participating in the study (CAAE 70984323.1.1001.5362) and registered with Clinicaltrials.gov (Clinical Trials identifier: NCT 05999526). The study protocol is available in the Supplementary Material .

Eligible participants were adults aged 18 years or older, admitted to the intensive care unit, intubated with an orotracheal tube, receiving MV for more than 72 hours, who underwent a successful SBT (as defined by the study protocol) and were deemed ready for extubation. Investigators randomized eligible participants immediately after a successful SBT to one of two groups: 1-hour positive pressure ventilation with ventilatory parameters as used before the SBT followed by extubation; or immediate extubation. The study protocol recommended for both groups daily assessment checklist to evaluate the eligibility for weaning and SBT;^( 3 – 5 )^ performing the SBT on pressure support or T-piece for 30 minutes;^( 6 )^ assessment and management of laryngeal edema risk;^( 7 , 8 )^ use of noninvasive ventilation and/or high flow nasal cannula for up to 24 hours after extubation in patients at high risk of extubation failure.^( 9 , 10 )^ As this was a study to assess feasibility (pilot), a convenience sample size of 60 patients was defined. Statistical analysis was described in the Supplementary Material .

## RESULTS

**Figure 1:**
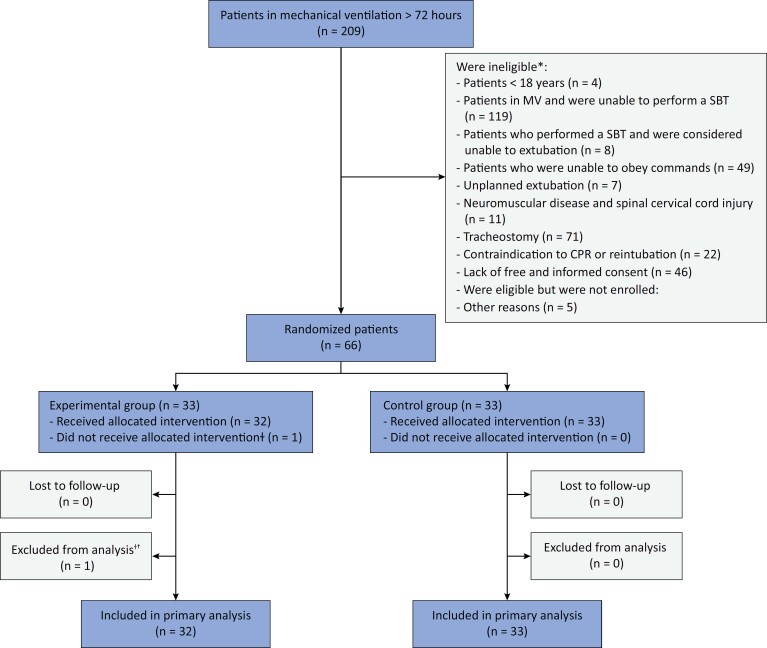
Enrollment, randomization, intervention, and follow-up.

From November 2023 to March 2024, 66 patients fulfilled eligibility criteria and were enrolled in the study well within the planned schedule ( Figure 1 ). One patient was excluded from the analysis due to ineligibility and incorrect randomization.

SBT - spontaneous breathing trial; CPR - cardiopulmonary resuscitation. * Each patient could meet more than one exclusion criteria; † one patient was randomized in error because he was not eligible and thus was excluded from the analysis. This patient was not extubated and was transferred to another hospital.

Table 1 describes the relevant baseline characteristics and outcomes. The study interventions were performed as assigned in 95% of patients (33 [100%] in the Control Group *versus* 30 [93.7%] in the Experimental Group). In the Experimental Group, two patients did not receive 1-hour positive pressure ventilation according to protocol but were included in the intention-to-treat analysis. Noninvasive ventilation and/or high-flow nasal cannula after extubation were used in 45.1% of patients of high-risk patients. Adherence to other protocol items was 100%. Extubation failure within 7 days occurred in 35% of patients (9 [28%] in the Experimental Group *versus* 14 [42.4%] in the Control Group). Additional results are available in figure 1S and tables 1S and 2S ( Supplementary Material ).

**Table 1:** Relevant baseline characteristics, feasibility outcomes, and clinical outcomes

			Experimental Group (n = 32)	Control Group (n = 33)	Difference (95%CI)
Relevant baseline characteristics					
	Weaning *				
		Short	22 (68.7)	21 (63.6)	-
		Difficult	10 (31.2)	11 (33.3)	-
		Prolonged	0 (0)	1 (3.0)	-
	Indication of orotracheal intubation				
		Surgical procedure	12 (37.5)	9 (27.2)	-
		Neurological	10 (31.2)	10 (30.3)	-
		Respiratory failure	9 (28.1)	10 (30.3)	-
		Circulatory	1 (3.1)	3 (9.0)	-
		Other	0 (0)	1 (3.0)	-
	SAPS 3		70 (61.5 - 78.5)	67 (57 - 80)	
	High risk of extubation failure		31 (96.8)	31 (94.0)	
	Very high risk of extubation failure †		11 (34.3)	8 (24.2)	
Feasibility outcomes					
	Primary outcome - adherence to mandatory items ‡		30 (93.7) §	33 (100)	-
	Adherence to suggested items				
		Checklist for assessing eligibility for weaning and SBT	32 (100)	33 (100)	-
		SBT, according to the protocol	32 (100)	33 (100)	-
		Assessment and management of laryngeal edema risk	32 (100)	33 (100)	-
		NIV and/or HFNC after extubation in high-risk patients	14/31 (45.1)	14/31 (45.1)	-
		NIV and/or HFNC duration (hours)	2.5 (2 - 3.5)	3 (2 - 4.5)	-
Clinical outcomes					
	Extubation failure within 7 days ¶		9 (28.1)	14 (42.4)	-14.4 (-35.3 to 8.5) ||
		SBT in pressure support	4/14 (28.5)	5/16 (31.2)	-
		SBT in T-piece	5/18 (27.7)	9/17 (53.0)	-
	Extubation failure – reasons				
		Respiratory failure	8/9 (88.8)	11/14 (78.5)	-
		Neurological	1/9 (11.1)	0/14 (0)	-
		Procedure	0/9 (0)	3/14 (21.5)	-
	MV free days at 28 days		28 (23 - 28)	28 (17 - 28)	1.52 (0.57 to 4.15) #
	ICU length of stay (days)		12.5 (6 - 17.5)	11 (8 - 20)	-
	Hospital length of stay (days)		26.5 (17.7 - 53.5)	24 (16 - 39)	-
	ICU mortality		2 (6)	5 (15)	-
	Hospital mortality		6 (19)	5 (15)	-

95%CI - 95% confidence interval; SAPS 3 - Simplified Acute Physiology Score 3; SBT - spontaneous breathing trial; NIV - noninvasive ventilation; HFNC - high flow nasal cannula; MV - mechanical ventilation; ICU - intensive care unit.

* Short weaning: the first separation attempt resulted in a termination of the weaning process within 24 hours; difficult weaning: weaning was terminated after more than 1 day but in less than 1 week after the first separation attempt; prolonged weaning: weaning was still not terminated 7 days after the first separation attempt;

† very high risk of extubation failure: with more than four high-risk criteria;

‡ feasibility: capability to complete the study according to the planned schedule and with adherence above 90% to the procedures of the experimental group (one-hour positive pressure ventilation for 1 hour ± 10 minutes after spontaneous breathing trial followed by extubation) and control group (extubation immediately after spontaneous breathing trial);

§ 1 patient required a bedside intervention immediately before extubation (surgical curative) - this patient was submitted to positive pressure ventilation for 130 minutes followed by extubation, and 1 patient was submitted to positive pressure ventilation but developed tachypnea and sweating and was not extubated;

¶ extubation failure was defined as reintubation or death within 7 days after randomization;

|| risk difference between a patient in the control group with extubation failure and a patient in the experimental group;

# odds of a patient in the control group having fewer mechanical ventilation-free days at 28 days than a patient in the experimental group. Results are expressed as n (%), median (interquartile range), or n/total n (%).

## DISCUSSION

These results demonstrate that it is feasible and needed to perform large, randomized trials to determine whether 1-hour positive pressure ventilation after a successful SBT, on T-piece or pressure support, reduces the risk of extubation failure within 7 days in patients who have undergone more than 72 hours of MV. Considering that the evidence for interventions involving SBTs using a T-piece is limited due to the small sample size of previous studies, and there is no trial regarding intervention during SBTs with pressure support, it is important to conduct a randomized trial to determine the effect of this intervention following a successful SBT using a T-piece and a separate trial after SBT on pressure support.

This study also tested the protocol, interventions, case report form, and data collection system. In addition, it identified areas to be improved in the large trials, such as the adherence to ventilatory support after extubation. The main differences between our feasibility trials and those of Fernandez et al. and Dadam et al.^( 1 , 2 )^ are presented in the Supplementary Material . If large clinical trials demonstrate a reduction in extubation failure within 7 days, the impact on critically ill patients could be significant, as extubation failure dramatically increases the risk of healthcare-associated infections (especially ventilator-associated pneumonia), length of hospital stay, mortality, and costs.^( 11 )^
