# Supplementary material for: One-hour positive pressure ventilation after a successful spontaneous breathing trial: a multicenter feasibility randomized clinical trial
Source: Crit Care Sci. 2025 May 4;37:e20250361. doi: 10.62675/2965-2774.20250361 (PMC12266821; doi:10.62675/2965-2774.20250361)
Supplement: Supplementary file 1 [file 2965-2774-ccsci-37-e20250361-Mat-suppl.pdf]

Critical Care Science
https://doi.org/10.62675/2965-2774.20250361

SUPPLEMENTARY MATERIAL

One-hour positive pressure ventilation after a successful spontaneous breathing trial: a multicenter feasibility randomized clinical trial

Aline Braz Pereira[1,2], Michelli Marcela Dadam[2], Bruna de Albuquerque Catelano[2], Daniela Delvan[2], Vitor Hugo Silva Pastorello[1], Luana Caroline Radun[1], Israel Silva Maia[3,4], Cassio Luis Zandonai[3], Eliana Vieira Santucci[4], Gabriela Souza Murizine[4], Marcelo Luiz Pereira Romano[4], Glauco Adrieno Westphal[1,2], Alexandre Biasi Cavalcanti[4]

STATISTICAL ANALYSIS AND RESULTS

Statistical analysis

Statistical analysis was conducted using the intention-to-treat principle. Continuous variables with non-normal distribution were described as median (interquartile range [IQR]). Adherence was presented as absolute frequencies and percentages. The effect of treatment on the extubation failure outcome was estimated as difference between proportions and 95% confidence interval. Kaplan-Meier curves were used to analyze the time to extubation failure outcome within 7 days, with hazard ratios and respective confidence intervals. The outcome of mechanical ventilation (MV) free days at 28 days was calculated using ordinal logistic regression with a 95% confidence interval.

Differences between our trial and the two trials published previously

Main differences between our trial and the two published previously:(1,2)

- In our trial, about half of the patients included underwent a successful spontaneous breathing trial (SBT) in pressure support ventilation.
- We defined extubation failure (secondary outcome in our feasibility trial, will be the primary outcome in the subsequent large trials) as reintubation or death within 7 days (according to WIND definition)(3) whereas the previous trials had reintubation within 48 hours as primary outcome.
- We have standardized assessment of readiness to weaning and SBT (with the aid of checklist), the actual SBT both in T-piece and pressure support, the use of noninvasive respiratory support (high-flow nasal oxygen or non-invasive ventilation) after extubation (mandatory for high-risk patients), assessment of risk of laryngeal edema and preventive use of corticosteroid.

REFERENCES

1. Dadam MM, Gonçalves ARR, Mortari GL, Klamt AP, Hippler A, Lago JU, et al. The Effect of Reconnection to Mechanical Ventilation for 1 Hour After Spontaneous Breathing Trial on Reintubation Among Patients Ventilated for More Than 12 Hours: A Randomized Clinical Trial. Chest. 2021;160(1):148-56.
2. Fernandez MM, González-Castro A, Magret M, Bouza MT, Ibañez M, García C, et al. Reconnection to mechanical ventilation for 1 h after a successful spontaneous breathing trial reduces reintubation in critically ill patients: a multicenter randomized controlled trial. Intensive Care Med. 2017;43(11):1660-7.
3. Béduneau G, Pham T, Schortgen F, Piquilloud L, et al. Epidemiology of Weaning Outcome according to a New Definition. The WIND Study. Am J Respir Crit Care Med. 2017 Mar 15;195(6):772-783.

RESULTS

Additional results are available in figure 1S and tables 1S and 2S.

This is an open access article under the CC BY license (https://creativecommons.org/licenses/by/4.0/).

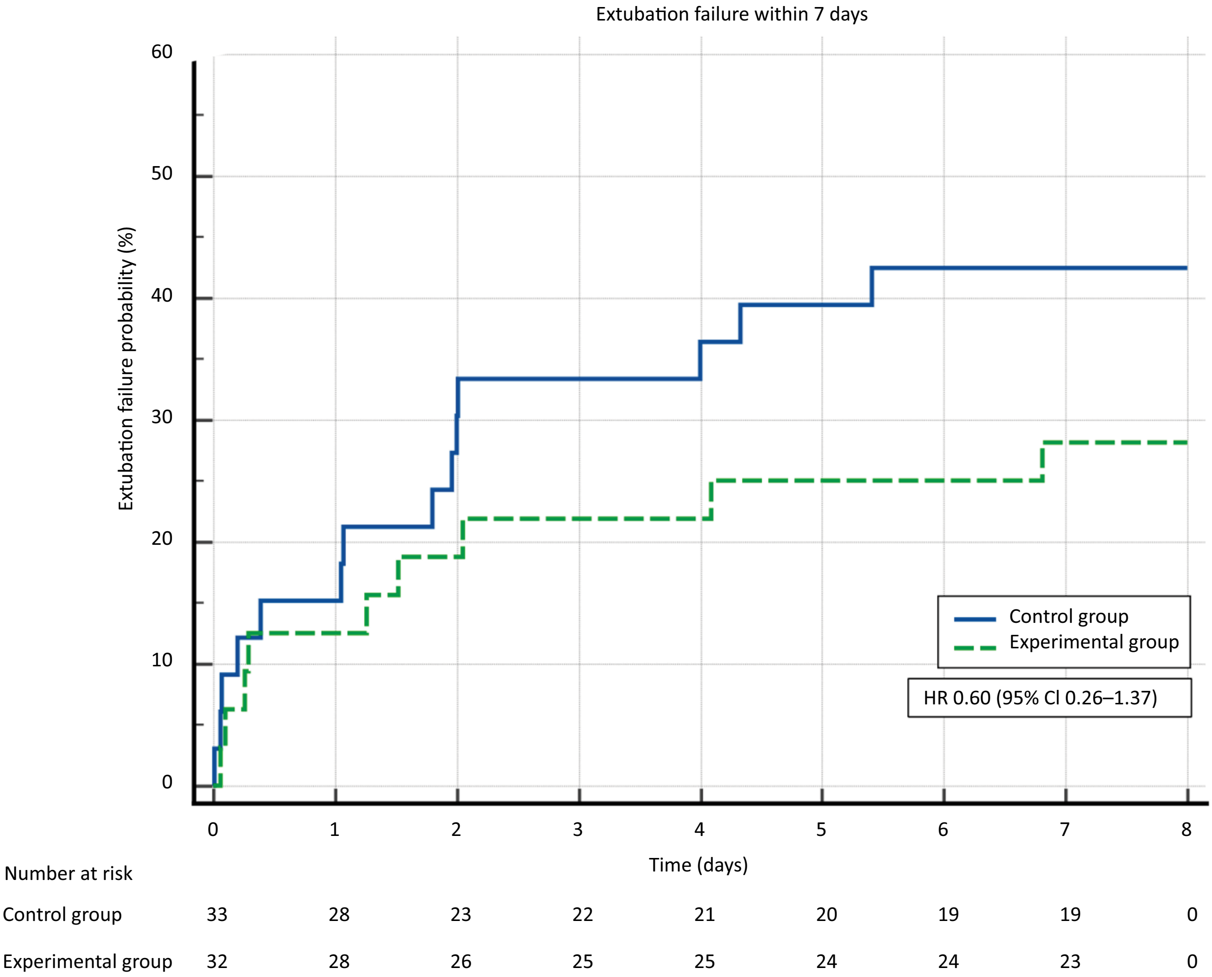

Figure 1S - Kaplan–Meier analysis of risk of extubation failure within 7 days after randomization in control vs experimental group.
Extubation failure was defined as reintubation or death within 7 days after randomization.

Table 1S - Baseline patient characteristics according to study group at randomization

Characteristic	Experimental Group (n = 32)	Control Group (n = 33)
Age (years)	63.9 (50.0 - 70.2)	62.1 (49.9 - 68.5)
Female sex	10 (31)	17 (51)
Comorbidities		
Systemic arterial hypertension	17 (53.1)	18 (54.5)
Diabetes Mellitus	8 (25.0)	8 (24.2)
Respiratory disease	6 (18.7)	2 (6.0)
Heart disease	6 (18.7)	5 (15.1)
Other diseases	6 (18.7)	7 (21.2)
Cancer	1 (3.1)	6 (18.1)

Continue...

...continuation

Neurological disease	3 (9.3)	3 (9.0)
Renal disease	1 (3.1)	3 (9.0)
Liver disease	1 (3.1)	1 (3.0)
Diagnosis at ICU admission		
Surgical	10 (31.2)	5 (15.1)
Neurological	9 (28.1)	8 (24.2)
Respiratory	6 (18.7)	9 (27.2)
Cardiovascular	4 (12.5)	2 (6.0)
Sepsis	3 (9.3)	5 (15.1)
Cancer	0 (0)	2 (6.0)
Liver disease	0 (0)	1 (3.0)
Renal/metabolic	0 (0)	1 (3.0)
Orotracheal intubation		
Non-elective	22 (68.7)	24 (72.7)
Elective	10 (31.2)	9 (27.2)
High-risk criteria		
MV > 7 days	17 (53.1)	15 (45.4)
BMI > 30kg/m^2	13 (40.6)	15 (45.4)
≥ 2 comorbities	13 (40.6)	16 (48.4)
Age > 65 years	13 (40.6)	12 (36.3)
> 1 failed SBT	12 (37.5)	13 (39.3)
Upper airway problems	10 (31.2)	18 (54.5)
COPD	2 (6.2)	1 (3.0)
Copious secretions	2 (6.2)	1 (3.0)
SAPS 3 > 50	0 (0)	0 (0)
MV time (days)	8.5 (5.4 - 11.5)	6.6 (4.4 - 8.7)
Previous attempts to SBT	0 (0 - 1.2)	0 (0 - 1)
Spontaneous breathing trial		
Pressure support	14 (43.7)	16 (48.4)
T-piece	18 (56.2)	17 (51.5)

ICU - intensive care unit; MV - mechanical ventilation; BMI - body mass index; SBT - spontaneous breathing trial; COPD - chronic obstructive pulmonary disease; SAPS-3 - Simplified Acute Physiology Score 3. Results expressed as median (interquartile range) or n (%).

Table 2S - Secondary outcomes

Outcome	Experimental Group (n = 32)	Control Group (n = 33)
Extubation failure within 48 hours*	6 (18.7)	10 (30.3)
Mortality - reasons		
Respiratory failure	0 (0)	1 (3)
Refractory shock	3 (9)	3 (9)
Multiple organ failure	3 (9)	0 (0)
Other	0 (0)	1 (3)

* Extubation failure was defined as reintubation or death within 48 hours after randomization.

STUDY PROTOCOL

Clinical Trial Registration: NCT 05999526.
Version: version 2.0; august 2023
CAAE: 70984323.1.1001.5362
Center coordinator: Centro Hospitalar Unimed de Joinville - Santa Catarina

Steering committee:
Aline Braz Pereira, MD
Glauco Adrieno Westphal, MD, PhD
Michelli Marcela Dadam, PT, MsC
Alexandre Biasi Cavalcanti, MD, PhD
Bruna de Albuquerque Catelano, PT, MsC
Vitor Hugo Silva Pastorello, PT, MsC
Luana Caroline Radun, RPh

Financing: The authors received no financial support for this study.

Corresponding author:
Aline Braz Pereira
Centro Hospitalar Unimed
Rua Orestes Guimarães, 905
Zip code: 89204-060 - Joinville (SC), Brazil
E-mail: linibp@hotmail.com

**Title**	Mechanical ventilation reconnection for one hour after spontaneous breathing trial: a randomized feasibility clinical trial
**Center coordinator**	Centro Hospitalar Unimed de Joinville - Santa Catarina
**Steering committee**	Aline Braz Pereira, MD<br>Glauco Adrieno Westphal, MD, PhD<br>Michelli Marcela Dadam, PT, MsC<br>Alexandre Biasi Cavalcanti, MD, PhD<br>Bruna de Albuquerque Catelano, PT, MsC<br>Vitor Hugo Silva Pastorello, PT, MsC<br>Luana Caroline Radun, RPh
**Desing**	This is a randomized, non-blinded, multicenter feasibility study (pilot) with intention-to-treat analysis. The study will compare two weaning strategies from MV in critically ill patients admitted to intensive care units, with more than 72 hours of MV and who had a successful SBT on pressure support or T-piece. Immediately after a successful SBT, eligible patients will be randomized into 2 groups that undergo the following interventions:<br>1) One-hour positive pressure ventilation: as soon as the success of the SBT is confirmed, the patient is submitted to mechanical ventilator for 1 hour using the previous ventilatory parameters and, afterwards, extubated.<br>2) Immediate extubation: the patient is extubated immediately after SBT.<br>Follow-up will be until hospital discharge or death.
**Primary objective**	Evaluate the feasibility of performing a large randomized controlled trial to determine whether one-hour positive pressure ventilation after a successful SBT reduces the risk of extubation failure within 7 days in patients with more than 72 hours of MV.<br>Feasibility is defined as the capability to complete the study according to the planned schedule and with adherence above 90% to the procedures of the experimental group (one-hour positive pressure ventilation [± 10 minutes] after SBT followed by extubation) and control group (extubation immediately after SBT).
**Inclusion criteria**	- Patients aged > 18 years.<br>- Admitted to the intensive care unit (surgical or medical).<br>- With orotracheal intubation.<br>- On MV for more than 72 hours.<br>- Who undergoes successful SBT (according to the study protocol) and is considered able to be extubated.
**Exclusion criteria**	- Patients unable t o obey commands.<br>- With non-planned extubation.<br>- Neuromuscular disease and cervical spinal cord injury.<br>- Tracheostomy.<br>- Contraindication for cardiopulmonary resuscitation or reintubation.<br>- Lack of informed consent.
**Treatment regimen**	One-hour positive pressure ventilation: as soon as the success of the SBT is confirmed, the patient is submitted to mechanical ventilator for 1 hour using the previous ventilatory parameters and, afterwards, extubated.
**Comparison regimen**	Immediate extubation: the patient is extubated immediately after a successful SBT.

Continue...

...continuation

**Follow-up**	Hospital discharge or death.
**Outcomes**	Primary outcome: Feasibility, defined as the capability to complete the study according to the planned schedule and with adherence above 90% to the procedures of the experimental group (one-hour positive pressure ventilation [± 10 minutes] after SBT followed by extubation) and control (extubation immediately after SBT).<br>Secondary outcomes:<br>- Extubation failure within 7 days, defined as a composite criterion of reintubation or death 7 days after randomization.<br>- Mechanical ventilation free days at 28 days.<br>- Intensive care unit length of stay<br>- Hospital length of stay<br>- Intensive care unit mortality.<br>- Hospital mortality.
**Sample size**	As this is a study to assess feasibility (pilot), a convenience sample size of 60 patients is defined, with planning to include 30 patients after SBT in pressure support and 30 patients after SBT in a T-piece.
**Statistical analysis**	Statistical analysis will be realized using the intention-to-treat principle. Continuous variables with non-normal distribution will be described as median (interquartile range [IQR]). Adherence will be described using absolute frequencies and percentages. The effect of treatment on the extubation failure outcome was estimated as difference between proportions and 95% confidence interval. Kaplan-Meier curves were used to analyze the time to extubation failure outcome within 7 days, with hazard ratios and respective confidence intervals. The outcome of MV free days at 28 days was calculated by ordinal logistic regression with respective 95% confidence interval.

INTRODUCTION

Extubation failure requiring reintubation occurs in 5-30% of patients and is associated with increased mortality.(1-7) The spontaneous breathing trial (SBT) is used to assess the patient tolerance for removal of invasive ventilatory support and to identify patients eligible for extubation.(8-15) Evidence suggests that a longer time to recover the minute-volume to baseline after the imposed work of the final SBT during one-hour positive pressure ventilation is associated with extubation failure, suggesting that ventilator disconnection for SBT may reduce the end expiratory lung volume and cause atelectasis.(16,17)

A physiological study demonstrated that SBT on pressure support, but principally T-piece, was associated with a significant reduction in end-expiratory lung volume and that one-hour positive pressure ventilation was associated with complete recovery of end-expiratory lung volume, regardless of the type of SBT.(18) Two randomized studies tested the hypothesis that one-hour positive pressure ventilation after a successful SBT would decrease extubation failure. Fernandez et al. demonstrated that one-hour positive pressure ventilation after successful SBT (most all with t-piece) likely reduces the reintubation rate within 48 hours in patients with more than 12 hours of MV.(19) Conversely, Dadam et al. showed that one-hour positive pressure ventilation after a successful SBT (with t-piece) did not reduce the risk of reintubation at 48 hours. However, the intervention might have been associated with a lower reintubation rate among patients with more than 72 hours of MV before extubation.(20)

These data suggest that the SBT causes loss of alveolar recruitment, and that one-hour positive pressure ventilation can promote recovery of lung volume to baseline levels before the start of SBT and consequently can reduce the rate of extubation failure in these patients. Larger studies are needed to confirm this hypothesis. Furthermore, no randomized controlled trials have assessed whether one-hour positive pressure ventilation is useful after a SBT on pressure support. The main goal of this study is to evaluate the feasibility of performing a large randomized controlled trial to determine whether one-hour positive pressure ventilation after a successful SBT, either on T-piece or pressure support, reduces the risk of extubation failure within 7 days in patients with more than 72 hours of MV.

Objectives

Primary objective

Assess the feasibility of performing a large randomized controlled trial to determine whether one-hour positive pressure ventilation after a successful SBT reduces the risk of extubation failure within 7 days in patients with more than 72 hours of MV.

Feasibility is defined as the capability to complete the study according to the planned schedule and with adherence above 90% to the procedures of the experimental group (one-hour positive pressure ventilation [± 10 minutes] after SBT followed by extubation) and control group (extubation immediately after SBT).

Secondary objective

Determine the effect of one-hour positive pressure ventilation after a successful spontaneous breathing trial in patients with more than 72 hours of MV on the following outcomes:

- Extubation failure, defined as a composite criterion of reintubation or death within 7 days after randomization.
- Mechanical ventilation free days at 28 days, defined as the number of days from the time of extubation and start of unassisted breathing until day 28 after randomization.
- Intensive care unit and hospital length of stay.
- Intensive care unit and hospital mortality.

METHODS

Study design

This is a randomized feasibility (pilot) non-blinded multicenter trial. Analysis will follow the intention-to-treat principle. The study will compare two weaning strategies from MV in critically ill patients admitted to intensive care units with more than 72 hours of MV and with a successful SBT on pressure support or T-piece. Immediately after a successful SBT, eligible participants will be randomized into 2 groups that undergo the following interventions:

1. One-hour positive pressure ventilation: as soon as the success of the SBT is confirmed, the patient will be submitted to one-hour positive pressure ventilation using the previous ventilatory parameters and, afterwards, extubated.

Previous ventilatory parameters are defined as the ventilatory mode in which the patient was being ventilated before the SBT (e.g., assist-control mode or pressure support) and the ventilatory parameters that include the eligibility criteria for the SBT, defined as $FiO_2 \leq 0.4$ and PEEP $\leq 8cmH_2O$.

In the experimental group, if signs of respiratory distress or other clinical problems occur during the one-hour positive pressure ventilation phase, the intensivist may decide to keep the patient on MV for more than 1 hour until reassessing the appropriate time for extubation.

2. Immediate extubation: the patient will be extubated immediately after SBT.

Follow-up will be until hospital discharge or death.

Study location

Four Brazilian sites will participate in the study.

Study population

Patients intubated on MV will be evaluated daily. Patients who met all the criteria below will be included:

Inclusion criteria (all must be present)

- Patients aged > 18 years;
- Admitted to the intensive care unit (surgical or medical);
- With orotracheal intubation;
- On MV for more than 72 hours;
- Who undergoes successful SBT (according to the study protocol) and is considered able to be extubated.

Exclusion criteria (none must be present)

- Patients unable to obey commands;
- Non-planned extubation;
- Neuromuscular disease and cervical spinal cord injury;
- Tracheostomy;
- Contraindication for cardiopulmonary resuscitation or reintubation;
- Lack of informed consent.

Pre-randomization care

All participating hospitals will use the same weaning protocol for the study. To ensure compliance with the

weaning protocol, a ventilator weaning checklist (Table 1S) will be applied daily at the bedside by the respiratory therapists to assess:[5, 6,12-15,19-23]

– The suitability for MV weaning;
– The eligibility for the SBT;
– The presence of SBT failure criteria.

Table 1S - Checklist for assessing eligibility for weaning and spontaneous breathing trial

**Eligibility for weaning from MV? (all must be present)**	
☐ Resolution of the acute phase of the disease that resulted in MV	
☐ Adequate oxygenation ($FiO_2 \leq 0,4$, $SpO_2 \geq 92\%$ e PEEP $\leq$ 8cmH$_2$O)	
**Eligible for spontaneous breathing trial?** Required items 1 to 4. Items 5 and 6 strongly suggested, but not required (depending on the institutional routine)	
☐ **1.** No sedation or minimal doses of sedative drugs	
☐ **2.** Capability to obey commands (at evaluator's criterion)	
☐ **3**. Absence or minimal doses of vasopressors (< 0.1mcg/kg/minute of noradrenaline)	
☐ **4.** No surgery scheduled within the subsequent 24 hours	
☐ **5.** Adequate cough (peak cough flow > 60L/minute assessed on mechanical ventilator or peak flow meter)	
☐ **6.** Low risk of post-extubation laryngeal edema or for high-risk patients and leak test with reduced leak volume, only after receiving intravenous methylprednisolone for at least 12 hours	
**Assessment and management of the risk of laryngeal edema:**<br>**1)** Is the patient at high risk of post-extubation laryngeal edema (patient with ≥ 72 hours of MV and at least one of the following criteria: female sex; difficulty to intubation; traumatic intubation; previous self-extubation)?<br>**2)** If YES, perform a volume leak test on mechanical ventilator<br>**3)** If leak test with leak volume < 24% of tidal volume or < 110mL, perform 20mg of intravenous methylprednisolone every 4 hours for 12 hours and postpone extubation for this period<br>If NO for the items above, proceed to start the SBT	
**After checking the steps above, start SBT for 30 minutes**	
**Spontaneous breathing trial?**	
☐ Support pressure (PEEP = 0cmH$_2$O e PS = 8cmH$_2$O)	☐ T-piece ($O_2 \leq$ 6L/minute)
**Presence of SBT failure signs?**<br>RR ≥ 35 breaths per minute; use of accessory muscles; paradoxical breathing; diaphoresis; HR > 140bpm, or sustained increase or decrease > 20%; systolic BP >180mmHg or < 80mmHg, or change > 20% in baseline value; lowering of the level of consciousness; psychomotor agitation.	
**If YES to any of the above, return to VM to rest**<br>**If NO, proceed with randomization for extubation**	
**Patients at high risk of extubation failure should receive NIV for 24 hours after extubation**<br>High risk of extubation failure is defined as a patient who presents one or more criteria: Age > 65 years; CHF as a cause of intubation; moderate or severe COPD; APACHE II > 12 or SAPS3 > 50 on the day of extubation; BMI > 30; presence of ≥ 2 comorbidities; ineffective cough or abundant secretion in the airways (> 2 aspirations in 8 hours before extubation); failure in more than one SBT; MV for > 7 days and; upper airway problems (including risk of developing laryngeal edema).	

FiO_2 - inspired fraction of oxygen; SpO_2 - peripheral oxygen saturation; PEEP - positive end expiratory pressure; MV - mechanical ventilation; SBT - spontaneous breathing trial; RR - respiratory rate; HR - heart rate; BP - blood pressure; CHF - congestive heart failure; COPD - chronic obstructive pulmonary disease; APACHE II - Acute Physiology and Chronic Health disease II; SAPS 3 - Simplified Acute Physiology Score III; BMI - body mass index.

Suitability for MV weaning and eligibility for the SBT is defined as:

- Resolution of the acute phase of the disease that resulted in MV;
- Adequate oxygenation ($FiO_2 \leq 0.4$, $SpO_2 \geq 92\%$, and PEEP $\leq 8cmH_2O$);
- No sedation or minimal doses of sedative drugs;
- Ability to follow commands (at the evaluator's discretion);
- Adequate cough (cough peak flow > 60L/minute assessed on a mechanical ventilator or peak flow meter);
- Absence or minimal doses of vasopressors (< 0.1mcg/kg/minute of norepinephrine);
- No scheduled surgery in the subsequent 24 hours;
- Low risk of post-extubation laryngeal edema or, for patients with a reduced cuff leak test volume, only after receiving intravenous methylprednisolone for at least 12 hours.

Measurement of cough peak flow

The measurement of cough peak flow can be performed using a mechanical ventilator or a peak flow meter. A value > 60 L/min will be considered adequate for MV weaning and eligibility for the SBT. Since the peak flow meter provides greater accuracy, the measurement on the mechanical ventilator should be used only if a peak flow meter is not available.[24-29]

- **Internal flow meter on the mechanical ventilator:** The ventilator settings should be the same as those used during the SBT. Instruct the patient to cough with maximum effort. At the same time, freeze the flow rate waveform. Visually select the peak flow rate on the graph and record the number to one decimal place. Repeat the measurements three consecutive times and record the best value. After the measurements, ventilate the patient with comfortable settings for 5 minutes for rest.[24]
- **Cough peak flow meter:** Disconnect the patient from the ventilator, connect the peak flow meter to the tracheal tube, and ask the patient to cough with maximum effort three consecutive times. Record the best of three attempts as the cough peak expiratory flow.[24,27,30]

Risk factors and diagnosis of post-extubation laryngeal edema

A patient with ≥ 72 hours of MV and presenting at least one of the following criteria will be considered at high risk for post-extubation laryngeal edema:[31-33]

- Female gender;
- Difficult intubation;
- Traumatic intubation;
- Previous self-extubation.

These patients will undergo a cuff leak test as described:

- Before performing the cuff leak test, first suction the endotracheal and oral secretions and set the ventilator to controlled mode;
- With the cuff inflated, record the inspiratory and expiratory tidal volumes displayed to ensure they are similar;
- Record the cuff pressure;
- Deflate the cuff;
- Record the expiratory tidal volume for the next six respiratory cycles, as the expiratory tidal volume will reach a plateau after a few cycles;
- Average the three lowest values;
- The difference between the inspiratory tidal volume (measured before cuff deflation) and the average expiratory tidal volume is the leak volume.[33-37]

Patients with a leak volume < 24% of the tidal volume or < 110 ml will receive 20 mg of intravenous methylprednisolone every 4 hours for 12 hours, and extubation will be delayed for this period.[32,33,37] For these patients, eligibility for the study and randomization will only be considered 12 hours after the recommended intervention for managing this specific case.

Spontaneous breathing trial

The SBT using pressure support will be performed without disconnecting the patient from the ventilator, using a low level of pressure support ($8cmH_2O$) with $FiO_2 \leq 40\%$ and no positive end-expiratory pressure (PEEP = 0). The T-piece SBT will be performed with the T-piece connected to the end of the tracheal tube, disconnected from the ventilator, and connected to a supplementary oxygen source (≤ 6L/minute).[22,23] Despite

studies showing no difference between SBT durations of 30 minutes compared to 120 minutes, the first 30 minutes are crucial in terms of discriminating between success and failure.[4,12,38] Therefore, we have opted to standardize and define the test duration as 30 minutes.

Failure of the SBT will be considered when the patient presents any of the following signs:

- Heart rate > 140 beats per minute or a sustained increase or decrease > 20%;
- Systolic blood pressure > 180mmHg or < 80mmHg or a change > 20% from baseline;
- SpO_2 < 90%;
- Respiratory rate ≥ 35 breaths per minute;
- Use of accessory muscles;
- Paradoxical breathing;
- Decreased level of consciousness;
- Diaphoresis.

The identification of any sign of intolerance during the SBT will be a criterion to immediately stop the test and return the patient to MV with previous ventilatory settings for rest.[12,13,19,20]

Randomization

Patients who meet the inclusion criteria will be randomized into two groups, which will undergo two different interventions:

1. One-hour positive pressure ventilation using the previous ventilatory parameters and, afterwards, extubated.
2. Immediate extubation.

Generation of the list

A randomization list will be generated using a statistical package. The randomization will be in variable-sized blocks and stratified by site and type of SBT (pressure support or T-piece). The randomization will be obtained through computer-generated random number tables in blocks of four for each hospital and will be unknown to the investigators involved in patient recruitment.

Randomization list concealment

Randomization will be centralized via the internet using appropriate software (REDCap). The group to which the patient will be allocated will only be disclosed after the information is entered into the electronic system. This prevents the investigator and medical team from predicting which treatment group the patient will be allocated to. Study data will be collected and managed using REDCap electronic data capture tools from HCor-Hospital do Coração.[39,40]

Allocation - Implementation

In the presence of an eligible patient, assistant respiratory therapists or intensivists will be instructed to call a study team member specifically responsible for randomization at each site. The randomization list will be generated by a statistician who will not participate in the study.

Blinding

Since the intervention will be administered to critically ill patients on MV, neither the patient nor the healthcare team or investigators will be blinded because this would not be feasible. As it is a non-pharmacological intervention, blinding the medical team is not feasible. There is no need for a committee to validate the study outcome (feasibility), and therefore, the outcome assessors will not be blinded.

Post-randomization interventions

Immediately after a successful SBT, eligible patients will be randomized into two groups that will undergo the following interventions:

1. One-hour positive pressure ventilation: as soon as the success of the SBT is confirmed, the patient is submitted to one-hour positive pressure ventilation using the previous ventilatory parameters and, afterwards, extubated.

Previous ventilatory parameters are defined as the ventilatory mode in which the patient was being ventilated before the SBT (e.g., assist-control mode or pressure support) and the ventilatory parameters that include the eligibility criteria for the SBT, defined as $FiO_2 \leq 0.4$ and PEEP ≤ $8cmH_2O$.

In the experimental group, if signs of respiratory distress or other clinical problems occur during the one-hour positive pressure ventilation phase, the intensivist may decide to keep the patient on MV for more than 1 hour until reassessing the appropriate time for extubation.

2. Immediate extubation: the patient will be extubated immediately after SBT.

All patients will be monitored by the respiratory therapist before, during, and after extubation. Adherence to

the checklist items will be a necessary condition to proceed with extubation.

Noninvasive ventilation and/or high-flow nasal cannula after extubation

The use of noninvasive ventilation and/or high-flow nasal cannula for 24 hours after extubation will be standardized, prescribed by the intensivist, to prevent reintubation in high-risk patients.[(41-44)] A patient will be considered at high risk for reintubation if they present one or more of the following criteria:

- Age > 65 years[(5,29,44)]
- Presence of congestive heart failure as the cause of intubation[(5,29,44-46)]
- Moderate or severe chronic obstructive pulmonary disease (COPD)[(45)]
- APACHE II > 12 or SAPS 3 > 50 on the day of extubation[(29,44)]
- Body mass index (BMI) > 30 (calculated as weight in kilograms divided by height in meters squared)[(47,48)]
- Presence of ≥ 2 comorbidities[(44)]
- Ineffective cough or abundant airway secretions (> 2 aspirations in 8 hours before extubation)[(44-46)]
- Failure in more than one SBT[(44)]
- Mechanical ventilation for > 7 days[(45,46)] and
- Upper airway problems (including risk of developing laryngeal edema)[(31,44)]

The high-flow nasal cannula should be applied immediately after extubation using a specific nasal cannula. The flow should initially be set to 10L/minute and titrated in increments of 5L/minute until the patient reports discomfort. The temperature should initially be set to 37°C unless discomfort is reported by the patient, and the FiO_2 should be regularly adjusted to maintain target SpO_2 > 92%. After 24 hours, high flow will be discontinued, and if necessary, patients will receive conventional oxygen therapy.[(31,49)]

Noninvasive ventilation with a full-face or oronasal mask will be provided immediately after extubation continuously for a scheduled period of 24 hours after extubation. Subsequently, non-invasive ventilation will be removed, and if necessary, patients will receive conventional oxygen therapy. Begin using a pressure support of $8cmH_2O$ and a PEEP of $5cmH_2O$.[(47)] Both PEEP and inspiratory pressure support will be adjusted to achieve a respiratory rate < 25/minute, tidal volume of 6 - 8mL/kg of predicted weight, and adequate gas exchange (SpO_2 ≥ 92% and pH > 7.35). FiO_2 will be adjusted to maintain SpO_2 ≥ 92%. Sedatives to enhance tolerance to non-invasive ventilation will not be allowed.[(31,50)] Patients with ≥ 4 risk factors, obesity, heart failure, or COPD benefit more from using non-invasive ventilation compared to high-flow nasal cannula, suggesting the use of non-invasive ventilation in this population.[(49-52)]

Noninvasive ventilation or high-flow nasal cannula should be used for at least 24 continuous hours, as tolerated by the patient, and can be used alternately with each other or with conventional oxygen therapy.

Extubation failure

Post-extubation acute respiratory failure with need of reintubation is defined by the occurrence of two of more of the following signs:[(12,19,20)]

- SpO_2 < 90%
- Tachypnea (respiratory ≥ 35 breaths per minute)
- Respiratory acidosis
- High respiratory effort (e.g. increased activity inspiratory accessory muscle, paradoxical breathing movements)
- Decreased level of consciousness
- Ineffective cough

In the presence of post-extubation acute respiratory failure signs, the clinician should strongly consider reintubation of the patient. There is no evidence of benefit from the use of non-invasive ventilation or high-flow nasal cannula in cases of acute respiratory failure due to extubation failure, as it may increase mortality by delaying intubation.[(53-56)]

In cases of extubation failure, if the patient requires another weaning attempt, they must follow the protocol and intervention to which they were originally randomized (one-hour positive pressure ventilation followed by extubation, or immediate extubation).

Interventions - Adherence

The protocol will be presented to doctors, nurses, and respiratory therapists through initial training sessions at study sites and meetings with the researchers every three months. Additionally, protocol compliance will be

assessed daily by the study coordinator, and feedback will be provided to the assisting team as needed. A doctor from the coordinating center will be available 24 hours a day to respond to specific questions or concerns that arise during the study. Data on adherence to the study protocol will be collected.

Concomitant interventions

Other patient care interventions are at the discretion of the teams at each institution.

Outcomes

Primary outcome

The primary outcome will be feasibility, defined as the capability to complete the study according to the planned schedule and with adherence above 90% to the procedures of the experimental group (rest for 1 hour [± 10 minutes] after SBT followed by extubation) and control group (extubation immediately after SBT).

Secondary outcomes

The secondary outcomes will be:

– Extubation failure, defined as a composite criterion of reintubation or death within 7 days after randomization. Weaning failure will be defined as failure within 7 days after randomization requiring reintubation and invasive MV, whether post-extubation non-invasive ventilation is used or not.(57) The composite outcome is chosen to account for patients who may die before 7 days without being reintubated. The choice of a 7-day outcome is based on the fact that the time interval to define extubation success varies in the literature, and evidence shows that many patients are reintubated after 48 - 72 hours, making these early times for assessing reintubation, especially in cases of prophylactic use of noninvasive ventilation or high-flow nasal cannula post-extubation.(23,50,58) If a patient is randomized to the experimental group, and after being submitted to one-hour positive pressure ventilation for any reason it is not extubated on the same day, it will be counted as an extubation failure

– Ventilator-free days in 28 days, defined as the number of days from the time of extubation and the start of unassisted breathing until day 28 after randomization. If a patient dies before day 28, the ventilator-free days will be counted as zero. If a patient is reintubated and returns to MV and later is extubated again and remains in unassisted breathing until day 28, the ventilator-free days will be counted from the end of the last period of assisted breathing until day 28. An invasive MV period of less than 24 hours for surgical purposes will count as 1 ventilator-free day. Patients discharged from the hospital in unassisted breathing before 28 days will be considered as ventilator-free for the remaining days up to 28 days. Patients transferred to another hospital or healthcare unit will be followed up until day 28 to assess this outcome(59,60)

– Length of stay in the intensive care unit

– Length of stay in the hospital

– Intensive care unit mortality

– Hospital mortality

Sample size calculation

This is a feasibility (pilot) study with a sample size defined by convenience, consisting of 60 patients. We intend to include 30 patients after SBT with pressure support and 30 patients after SBT with a T-piece.

Recruitment

The medical and respiratory therapist team in the intensive care unit will recruit patients with more than 72 hours of MV who are suitable for weaning through the daily bedside application of the ventilator weaning checklist (Table 1S). We expect a recruitment rate of 3 patients per month per site over a period of 6 months.

Study variables and visits

Data will be collected bedside by the respiratory therapist's teams of the participating hospitals, recorded on a paper form, and transferred to an electronic form. Researchers will be trained for this specific task before the study begins. Only trained and authorized researchers will have access to the study forms.

The study visits and the variables collected at each visit are described below and in table 2S. It is extremely important that investigators complete the forms in a timely manner. The deadline for completing the baseline, treatment, and follow-up forms is two days after the respective date (for example, no later than two days after the randomization date for baseline data). The deadline for completing the termination forms is 7 days after the respective date.

Table 2S - Study visits

	Study duration		
	Screening and allocation	Follow-up	Termination
**Time**	**Day 0**	**Day 1 - Day 7**	**Day 28 - Day 90**
Recruitment			
Consent form	X		
Spontaneous breathing trial	X		
Randomization	X		
Interventions			
Experimental group	X		
Control group	X		
Assessments			
Baseline variables list	X		
Outcome variables list	X	X	X

Patients will be followed until death or hospital discharge. For patients hospitalized for more than 90 days after randomization, follow-up will be truncated at 90 days. In these cases, patients will be considered to have been discharged alive.

Screening (Day 0)

- Weaning readiness: Resolution of the acute phase of the disease that led to MV and adequate oxygenation ($FiO_2 \leq 0.4$, $SpO_2 \geq 92\%$, and PEEP $\leq 8cmH_2O$).
- SBT readiness: No sedation or minimal doses of sedative drugs, ability to follow commands, adequate cough (cough peak flow > 60L/minute assessed with peak flow meter or mechanical ventilator), absence of or minimal doses of vasopressors (< 0.1mcg/kg/min of norepinephrine), no scheduled surgery in the next 24 hours.
- Evaluation of laryngeal edema risk and management according to protocol: Patient with ≥ 72 hours of MV and at least one of the following criteria: female sex; difficult intubation; traumatic intubation; previous self-extubation.
- Absence of SBT failure criteria: Respiratory rate ≥ 35 breaths per minute, use of accessory muscles, paradoxical breathing, diaphoresis, heart rate > 140bpm, or a sustained increase or decrease > 20%, systolic blood pressure > 180mmHg or < 80mmHg, or a change > 20% from baseline value, decreased level of consciousness.
- Inclusion criteria: Age ≥ 18 years, intensive care unit admission, orotracheal intubation and MV for more than 72 hours, who undergoes successful SBT (according to the study protocol) and is considered able to be extubated.
- Reason for not randomizing eligible patients: Unable to follow commands, unplanned extubation, neuromuscular disease and spinal cord injury, tracheostomy, contraindication for cardiopulmonary resuscitation or reintubation, refusal to sign informed consent.

Baseline data and randomization (Day 0):

Randomization:

- Date and time
- Spontaneous breathing trial: pressure support or T-piece

Baseline data:

- Birth sex
- Height
- Weight (reported by family or estimated if no scale available)
- Date of birth
- Hospital admission date
- Intensive care unit admission date

- SAPS-3
- Intensive care unit admission diagnosis: cardiovascular, respiratory, neurological, neoplasm, hepatic disease, renal/metabolic, surgical, sepsis
- Date and time of orotracheal intubation
- Orotracheal intubation: elective or non-elective
- Indication for orotracheal intubation: respiratory failure, neurological, circulatory, procedure, other
- Number of previous SBT attempts
- Risk of extubation failure: age > 65 years, presence of congestive heart failure as the cause of intubation, moderate or severe COPD, APACHE II > 12 or SAPS3 > 50 on the day of extubation, BMI > 30, presence of ≥ 2 comorbidities, ineffective cough or abundant airway secretions (> 2 aspirations in 8 hours before extubation), failure in more than one SBT, MV for > 7 days

Comorbidities (Charlson):[61-63]

- Hypertension:

Patients without diabetes or renal disease with systolic pressure > 140mmHg and/or diastolic pressure > 90mmHg, patients with controlled hypertension, and patients with diabetes or renal disease with systolic pressure > 140mmHg and/or diastolic pressure > 80mmHg.

- Heart disease:

Myocardial infarction: Defined as one or more probable or confirmed myocardial infarctions. Patients hospitalized for chest pain or an equivalent clinical event with electrocardiographic and/or enzymatic changes. Patients with isolated electrocardiographic changes without clinical signs of infarction will not be considered as having an infarction.

Hospitalization or treatment for heart failure: Defined as congestive heart failure. Patients with paroxysmal nocturnal dyspnea or exertional dyspnea whose symptoms respond to digitalis, diuretics, or afterload-reducing agents (or improved physical examination after taking one of these medications). Patients who do not respond and show no evidence of improvement in physical signs with treatment will not be considered as having heart failure.

Angina: Patients with chronic exertional angina, with a history of myocardial revascularization, and those initially admitted with unstable angina.

Arrhythmia: Patients with chronic atrial fibrillation or flutter, sinus node disease, or ventricular arrhythmias requiring chronic treatment.

Valvular disease: Patients with hemodynamically significant aortic stenosis and/or regurgitation, significant mitral stenosis and/or regurgitation, aortic or mitral valve prosthesis, hypertrophic obstructive cardiomyopathy requiring treatment, or tricuspid regurgitation.

Cardiogenic shock or cardiopulmonary resuscitation: Patients with these events before intensive care unit admission.

- Peripheral vascular disease:

Patients with intermittent claudication, history of bypass for arterial insufficiency, gangrene or acute arterial insufficiency, and those with a thoracic or abdominal aneurysm measuring 6 cm or more, treated or untreated.

- Neurological disease:

Stroke or transient ischemic attack: Including patients with minor symptoms or no residual symptoms.

Hemiplegia: Patients with hemiplegia or paraplegia resulting from a stroke or other conditions.

Alzheimer's disease, dementia of any cause, or severe cognitive impairment: Patients with moderate to severe chronic cognitive deficits resulting in impaired function, regardless of the cause.

Other neurological conditions: Patients with Parkinson's disease, seizures, or unexplained syncope.

- Respiratory disease:

COPD: Patients diagnosed with COPD with continuous symptoms such as dyspnea or cough with light or moderate activity. Including patients with dyspnea with light activity with or without treatment; with dyspnea with moderate activity despite treatment; with dyspnea at rest despite treatment; requiring continuous oxygen; with CO_2 retention; and those with baseline PO_2 below 50 torr.

Asthma: Patients diagnosed with asthma with continuous symptoms such as dyspnea or cough with light or moderate activity. Including patients with dyspnea with light activity with or without treatment, dyspnea with moderate activity despite treatment, and dyspnea at rest despite treatment.

Other respiratory conditions: Patients with interstitial lung disease, restrictive lung disease, pulmonary embolism, vascular disease, or severe pulmonary hypertension (> 40mmHg) of any cause resulting in severe exercise restriction (e.g., unable to climb stairs or perform household tasks).

Smoking habit: Active smokers consuming > 10 cigarettes/day with > 10 pack-years.

– Diabetes Mellitus:

All patients with diabetes treated with insulin or hypoglycemic agents, but not those treated with diet alone. Patients with gestational diabetes will not be considered diabetic. This classification also includes patients with target organ damage (retinopathy, neuropathy, nephropathy) attributable to diabetes.

– Renal disease:

Patients with moderate renal insufficiency, with creatinine > 3mg/dL. Severe renal disease including patients on dialysis, transplant recipients, and those with uremia.

– Liver disease:

Patients with mild liver disease (chronic hepatitis B or C or cirrhosis without portal hypertension), moderate liver disease (cirrhosis with portal hypertension but no bleeding), and severe liver disease (ascites, chronic jaundice, portal hypertension, history of variceal bleeding, or transplant).

– Cancer:

Lymphoma: Patients with Hodgkin's disease, lymphosarcoma, Waldenstrom's macroglobulinemia, myeloma, or other lymphomas.

Leukemia: Patients with acute or chronic myeloid leukemia, acute or chronic lymphocytic leukemia, or polycythemia vera.

Solid organ tumor: Patients with solid tumors without documented metastases, including breast, colon, lung, prostate, melanoma, and a variety of other tumors.

Metastatic cancer: Patients with metastatic solid tumors, including the same sites as detailed above.

– Other diseases:

Peptic ulcer disease: Patients requiring treatment for gastric or peptic ulcer, including those who had upper gastrointestinal bleeding due to peptic ulcer.

Rheumatic or connective tissue disease: Patients with systemic lupus erythematosus, polymyositis, mixed connective tissue disease, rheumatoid arthritis, polymyalgia rheumatica, vasculitis, sarcoidosis, Sjögren's syndrome, or any systemic vasculitis.

HIV (human immunodeficiency virus) or AIDS (acquired immunodeficiency syndrome): Patients with defined or probable AIDS, i.e., prodrome of HIV infection, as well as asymptomatic HIV-positive patients.

Decubitus ulcers, peripheral skin ulcers, or repeated episodes of cellulitis: Partial thickness skin loss on legs or back with open ulcers, or two or more episodes of cellulitis requiring antibiotic treatment, regardless of etiology.

Depression: Patients receiving treatment for depression, either pharmacological or psychotherapeutic, and those with signs indicating probable or confirmed depression.

Coagulopathy: Patients with coagulation disorders or using anticoagulants for any medical condition.

Other endocrine diseases: Patients with hypopituitarism, adrenal insufficiency, or recurrent acidosis.

Inflammatory bowel disease: Patients with ulcerative colitis, Crohn's disease, or regional enteritis.

Gastrointestinal bleeding: Patients with bleeding requiring transfusions for causes other.

Intervention (Day 0):

- Date and time of extubation.
- Use of non-invasive ventilation after extubation, duration.
- Assessment and management of the risk of laryngeal edema according to the protocol.
- Perform the SBT according to protocol.

Outcomes:

- Reintubation or death, date and time.
- Indication for orotracheal reintubation: respiratory failure, neurological, circulatory, procedure, other.
- Place of death (intensive care unit or ward), cause (respiratory failure/refractory hypoxemia, refractory shock, multiple organ failure, severe arrhythmia, unknown, other).
- Date of intensive care unit discharge.
- Date of hospital discharge.
- Mechanical ventilation free days in 28 days - weaning status from Day 1 to 28: extubated, orotracheal intubation with invasive MV, tracheostomy invasive MV, tracheostomy without invasive MV.

Statistical analysis

Statistical analysis will be realized using the intention-to-treat principle. Continuous variables with non-normal distribution will be described as median (interquartile

range [IQR]). Adherence will be described using absolute frequencies and percentages. The effect of treatment on the extubation failure outcome will be estimated as difference between proportions and 95% confidence interval. Kaplan-Meier curves will be used to analyze the time to extubation failure outcome within 7 days, with hazard ratios and respective confidence intervals. The outcome of MV free days at 28 days will be calculated by ordinal logistic regression with respective 95% confidence interval.

SCHEDULE

The schedule is presented in table 3S.

Table 3S - Schedule

	1	2	3	4	5	6	7	8	9	10	11	12	13	14	15
Evaluation by ethics committees	■	■	■												
Case report form and database		■	■	■											
Training of sites				■	■										
Inclusion of patients				■	■	■	■	■	■	■					
Follow-up of patients				■	■	■	■	■	■	■	■				
Data analysis												■	■		
Writing and submission of the paper													■	■	■

ETHICAL ASPECTS

The study will be conducted in accordance with national and international resolutions described in the following documents:

Resolution nº 196, dated October 10, 1996, and complementary ordinances of the National Health Council/Ministry of Health.

Declaration of Helsinki and all its revisions and amendments.

Study approval

Before starting the study, the investigator must submit a copy of the protocol, a copy of the informed consent form, and other necessary declarations to the Ethics Committee of each participating institution. A cover letter and an approval letter from the Ethics Committee, if obtained, must be sent to the Coordinating Center. Additionally, all protocol amendments must be approved by the Ethics Committee of each participating site.

Informed consent

Written consent will be sought from the legal representative of eligible patients because their clinical condition (MV, sedation) does not allow them to provide consent directly. The Responsible Researcher or Study Coordinator will be responsible for obtaining consent and providing the legal representative with information about the study. The patient's legal representative and the investigator responsible for obtaining consent must date and sign two copies of the informed consent form. One copy will be given to the patient's legal representative, and the other will be filed with the study documents. The investigator will clearly explain that participation is voluntary and that the patient or their legal representative may withdraw consent and leave the study at any time without any consequences for the quality and management of subsequent medical treatment. The proposed informed consent form must be reviewed by each research site; any necessary changes must be approved by the Study Coordinating Center before being submitted to the Ethics Committee.

Criteria for withdrawing patients from the study

Withdrawal from the study will only occur in cases where informed consent is withdrawn by the patient, their legal representative, or the patient's doctor.

Data confidentiality

Patient identification data will not be sent to the Study Coordinating Center. Each patient and research site will be identified in electronic form by a unique number. Information obtained from medical records must be treated as confidential data by research sites; it must be stored in restricted-access locations and anonymity must be ensured in interim and final reports.

Progress report

Investigators must submit written summaries of the study's status to the Ethics Committee of their institution every six months, as well as a final report at the end of the study.

Declaration of interest

There will be no funding source or conflict of interest from the study researchers.

Study organization

Coordinating Center

The study will be conducted by a team from the Unimed Hospital Center in Joinville, Santa Catarina. The Coordinating Center team includes Principal Investigator Aline Braz Pereira. The Coordinating Center is responsible for:

- Planning and conducting the study
- Designing the protocol
- Designing the electronic case report forms (e-CRF)
- Designing the operating guide
- Managing and controlling data quality
- Designing, testing, and maintaining the electronic data capture system
- Continuous data quality control
- Assisting the Steering Committee
- Managing the research sites
- Selecting and training research sites
- Helping sites prepare a regulatory report to be submitted to Ethics Committee and assisting sites with submission
- Monitoring recruitment rates and actions to increase recruitment
- Follow-up and implementation of actions to prevent follow-up losses
- Sending study materials to research sites
- Developing support materials for the study
- Statistical analysis and research reports
- Complete statistical analysis
- Helping to write the final manuscript

Steering Committee

The Steering Committee is responsible for the overall supervision of the study, assisting in the development of the study protocol and preparation of the final manuscript. All other study committees report to the Steering Committee. The members of the Steering Committee are trained investigators in the design and conduct of randomized clinical trials, intensivists, and respiratory therapists experienced in conducting multicenter randomized studies on MV weaning. The members of the Steering Committee are:

- Aline Braz Pereira, MD. Hospital Municipal São José, Joinville (SC), Unimed Hospital Center, Joinville (SC).
- Glauco Adrieno Westphal, MD, PhD. Hospital Municipal São José, Joinville (SC), Unimed Hospital Center, Joinville (SC).
- Michelli Marcela Dadam, PT, MSc. Hospital Municipal São José, Joinville (SC).
- Alexandre Biasi Cavalcanti, MD, PhD. Epidemiologist, intensivist. Coordinator of Research Initiatives at IEP-HCor, São Paulo (SP).
- Bruna de Albuquerque Catelano, PT, MSc. Hospital Municipal São José, Joinville (SC).
- Vitor Hugo Silva Pastorello, PT, MSc. Unimed Hospital Center, Joinville (SC).
- Luana Caroline Radun, RPh. Unimed Hospital Center, Joinville (SC).

Study sites

Four hospitals will be invited to participate in the study. Considering a monthly inclusion rate of 3 patients per site, the estimated patient inclusion period is approximately 6 months.

Publication policy

The study's Steering Committee is responsible for publishing the study results, regardless of the outcomes. As this is a randomized, collaborative, large-scale study, we intend to submit the main manuscript to high-impact journals. The success of the study will depend on the team and a collaborative effort among researchers, research coordinators, and patients. Therefore:

– The names of all researchers will be listed at the end of the manuscripts or as supplementary material, depending on each journal's editorial policy. The names will be listed in alphabetical order by site name.

– Suggestions for sub-study topics and secondary publications should be submitted by researchers to the Steering Committee, which will evaluate the proposal and may approve, suggest improvements, or reject it. The evaluation will be based on scientific merit and the researchers' contribution to the success of the main study.

Potential benefits of the study

The large clinical trial, which we intend to conduct following this pilot study, is being designed to address an important question in the care of critically ill patients with absolutely solid methodology. The study will produce a high-quality effect estimate of the impact of the therapeutic intervention on the risk of extubation failure. The hypothesis that one-hour positive pressure ventilation after a successful SBT reduces extubation failure is highly plausible based on physiological principles and evidence from two previous randomized studies. Therefore, if the results of the large clinical trial demonstrate a reduction in extubation failure within 7 days, the relevance for critically ill patients is significant, as extubation failure greatly increases the risk of healthcare-associated infections (especially ventilator-associated pneumonia), length of hospital stay, mortality, and costs. Ultimately, the study may contribute to improved care for critically ill patients and a high-quality healthcare system.

BUDGET

The costs of materials for conducting the study will be covered by the researchers' own resources.

REFERENCES

1. MacIntyre N. Discontinuing mechanical ventilatory support. Chest. 2007;132(3):1049-56.
2. Frutos-Vivar F, Esteban A, Apezteguia C, González M, Arabi Y, Restrepo MI, et al. Outcome of reintubated patients after scheduled extubation. J Crit Care. 2011;26(5):502-9.
3. Epstein SK, Ciubotaru RL, Wong JB. Effect of failed extubation on the outcome of mechanical ventilation. Chest. 1997;112(1):186-92.
4. Esteban A, Alía I, Tobin MJ, Gil A, Gordo F, Vallverdú I, et al.; Spanish Lung Failure Collaborative Group. Effect of spontaneous breathing trial duration on outcome of attempts to discontinue mechanical ventilation. Am J Respir Crit Care Med. 1999;159(2):512-8.
5. Thille AW, Harrois A, Schortgen F, Brun-Buisson C, Brochard L. Outcomes of extubation failure in medical intensive care unit patients. Crit Care Med. 2011;39(12):2612-8.
6. Peñuelas Ó, Thille AW, Esteban A. Discontinuation of ventilatory support: new solutions to old dilemmas. Curr Opin Crit Care. 2015;21(1):74-81.
7. Jaber S, Quintard H, Cinotti R, Asehnoune K, Arnal JM, Guitton C, et al. Risk factors and outcomes for airway failure versus non-airway failure in the intensive care unit: a multicenter observational study of 1514 extubation procedures. Crit Care. 2018;22(1):236.
8. Esteban A, Frutos F, Tobin MJ, Alía I, Solsona JF, Valverdú I, et al. A comparison of four methods of weaning patients from mechanical ventilation. Spanish Lung Failure Collaborative Group. N Engl J Med. 1995;332(6):345-50.
9. Tobin MJ, Perez W, Guenther SM, Semmes BJ, Mador MJ, Allen SJ, et al. The pattern of breathing during successful and unsuccessful trials of weaning from mechanical ventilation. Am Rev Respir Dis. 1986;134(6):1111-8.
10. DeHaven CB Jr, Hurst JM, Branson RD. Evaluation of two different extubation criteria: attributes contributing to success. Crit Care Med. 1986;14(2):92-4.
11. Millbern SM, Downs JB, Jumper LC, Modell JH. Evaluation of criteria for discontinuing mechanical ventilatory support. Arch Surg. 1978;113(12):1441-3.
12. Boles JM, Bion J, Connors A, Herridge M, Marsh B, Melot C, et al. Weaning from mechanical ventilation. Eur Respir J. 2007;29(5):1033-56.
13. MacIntyre NR, Cook DJ, Ely EW Jr, Epstein SK, Fink JB, Heffner JE, et al.; American College of Chest Physicians; American Association for Respiratory Care; American College of Critical Care Medicine. Evidence-based guidelines for weaning and discontinuing ventilatory support: a collective task force facilitated by the American College of Chest Physicians; the American Association for Respiratory Care; and the American College of Critical Care Medicine. Chest. 2001;120(6 Suppl):375S-95S.
14. Girard TD, Alhazzani W, Kress JP, Ouellette DR, Schmidt GA, Truwit JD, et al.; ATS/CHEST Ad Hoc Committee on Liberation from Mechanical Ventilation in Adults. An Official American Thoracic Society/American College of Chest Physicians Clinical Practice Guideline: Liberation from Mechanical Ventilation in Critically Ill Adults. Rehabilitation Protocols, Ventilator Liberation Protocols, and Cuff Leak Tests. Am J Respir Crit Care Med. 2017;195(1):120-33.
15. Schönhofer B, Geiseler J, Dellweg D, Fuchs H, Moerer O, Weber-Carstens S, et al. Prolonged weaning: S2k Guideline published by the German Respiratory Society. Respiration. 2020:1-102.

16. Martinez A, Seymour C, Nam M. Minute ventilation recovery time: a predictor of extubation outcome. Chest. 2003;123(4):1214-21.
17. Hernandez G, Fernandez R, Luzon E, Cuena R, Montejo JC. The early phase of the minute ventilation recovery curve predicts extubation failure better than the minute ventilation recovery time. Chest. 2007;131(5):1315-22.
18. Coudroy R, Lejars A, Rodriguez M, Frat JP, Rault C, Arrivé F, et al. Physiologic effects of reconnection to the ventilator for 1 hour following a successful spontaneous breathing trial. Chest. 2024;165(6):1406-14.
19. Fernandez MM, González-Castro A, Magret M, Bouza MT, Ibañez M, García C, et al. Reconnection to mechanical ventilation for 1 h after a successful spontaneous breathing trial reduces reintubation in critically ill patients: a multicenter randomized controlled trial. Intensive Care Med. 2017;43(11):1660-7.
20. Dadam MM, Gonçalves AR, Mortari GL, Klamt AP, Hippler A, Lago JU, et al. The effect of reconnection to mechanical ventilation for 1 hour after spontaneous breathing trial on reintubation among patients ventilated for more than 12 hours: a randomized clinical trial. Chest. 2021;160(1):148-56.
21. Epstein SK. Weaning from ventilatory support. Curr Opin Crit Care. 2009;15(1):36-43.
22. Subirà C, Hernández G, Vázquez A, Rodríguez-García R, González-Castro A, García C, et al. Effect of pressure support vs T-piece ventilation strategies during spontaneous breathing trials on successful extubation among patients receiving mechanical ventilation: a randomized clinical trial. JAMA. 2019;321(22):2175-82.
23. Thille AW, Gacouin A, Coudroy R, Ehrmann S, Quenot JP, Nay MA, et al.; REVA Research Network. Spontaneous-breathing trials with pressure-support ventilation or a T-piece. N Engl J Med. 2022;387(20):1843-54.
24. Bai L, Duan J. Use of cough peak flow measured by a ventilator to predict re-intubation when a spirometer is unavailable. Respir Care. 2017;62(5):566-71.
25. Smailes ST, McVicar AJ, Martin R. Cough strength, secretions and extubation outcome in burn patients who have passed a spontaneous breathing trial. Burns. 2013;39(2):236-42.
26. Salam A, Tilluckdharry L, Amoateng-Adjepong Y, Manthous CA. Neurologic status, cough, secretions and extubation outcomes. Intensive Care Med. 2004;30(7):1334-9.
27. Smina M, Salam A, Khamiees M, Gada P, Amoateng-Adjepong Y, Manthous CA. Cough peak flows and extubation outcomes. Chest. 2003;124(1):262-8.
28. Duan J, Zhang X, Song J. Predictive power of extubation failure diagnosed by cough strength: a systematic review and meta-analysis. Crit Care. 2021;25(1):357.
29. Ferrer M, Valencia M, Nicolas JM, Bernadich O, Badia JR, Torres A. Early noninvasive ventilation averts extubation failure in patients at risk: a randomized trial. Am J Respir Crit Care Med. 2006;173(2):164-70.
30. Abedini M, Froutan R, Bagheri Moghaddam A, Mazloum SR. Comparison of "cough peak expiratory flow measurement" and "cough strength measurement using the white card test" in extubation success: a randomized controlled trial. J Res Med Sci. 2020;25:52.
31. Hernández G, Vaquero C, Colinas L, Cuena R, González P, Canabal A, et al. Effect of postextubation high-flow nasal cannula vs noninvasive ventilation on reintubation and postextubation respiratory failure in high-risk patients: a randomized clinical trial. JAMA. 2016;316(15):1565-74.
32. Cheng KC, Hou CC, Huang HC, Lin SC, Zhang H. Intravenous injection of methylprednisolone reduces the incidence of postextubation stridor in intensive care unit patients. Crit Care Med. 2006;34(5):1345-50.
33. François B, Bellissant E, Gissot V, Desachy A, Normand S, Boulain T, et al.; Association des Réanimateurs du Centre-Ouest (ARCO). 12-h pretreatment with methylprednisolone versus placebo for prevention of postextubation laryngeal oedema: a randomised double-blind trial. Lancet. 2007;369(9567):1083-9.
34. Jaber S, Chanques G, Matecki S, Ramonatxo M, Vergne C, Souche B, et al. Post-extubation stridor in intensive care unit patients. Risk factors evaluation and importance of the cuff-leak test. Intensive Care Med. 2003;29(1):69-74.
35. Kuriyama A, Jackson JL, Kamei J. Performance of the cuff leak test in adults in predicting post-extubation airway complications: a systematic review and meta-analysis. Crit Care. 2020;24(1):640.
36. Miller RL, Cole RP. Association between reduced cuff leak volume and postextubation stridor. Chest. 1996;110(4):1035-40.
37. Fan T, Wang G, Mao B, Xiong Z, Zhang Y, Liu X, et al. Prophylactic administration of parenteral steroids for preventing airway complications after extubation in adults: meta-analysis of randomised placebo controlled trials. BMJ. 2008;337:a1841.
38. Vallverdú I, Calaf N, Subirana M, Net A, Benito S, Mancebo J. Clinical characteristics, respiratory functional parameters, and outcome of a two-hour T-piece trial in patients weaning from mechanical ventilation. Am J Respir Crit Care Med. 1998;158(6):1855-62.
39. Harris PA, Taylor R, Thielke R, Payne J, Gonzalez N, Conde JG. Research electronic data capture (REDCap)—a metadata-driven methodology and workflow process for providing translational research informatics support. J Biomed Inform. 2009;42(2):377-81.
40. Harris PA, Taylor R, Minor BL, Elliott V, Fernandez M, O'Neal L, et al.; REDCap Consortium. The REDCap consortium: building an international community of software platform partners. J Biomed Inform. 2019;95:103208.
41. Ni YN, Luo J, Yu H, Liu D, Liang BM, Yao R, et al. Can high-flow nasal cannula reduce the rate of reintubation in adult patients after extubation? A meta-analysis. BMC Pulm Med. 2017;17(1):142.
42. Xu Z, Li Y, Zhou J, Li X, Huang Y, Liu X, et al. High-flow nasal cannula in adults with acute respiratory failure and after extubation: a systematic review and meta-analysis. Respir Res. 2018;19(1):202.
43. Zhu Y, Yin H, Zhang R, Ye X, Wei J. High-flow nasal cannula oxygen therapy versus conventional oxygen therapy in patients after planned extubation: a systematic review and meta-analysis. Crit Care. 2019 May;23(1):180.
44. Nava S, Gregoretti C, Fanfulla F, Squadrone E, Grassi M, Carlucci A, et al. Noninvasive ventilation to prevent respiratory failure after extubation in high-risk patients. Crit Care Med. 2005;33(11):2465-70.
45. Thille AW, Boissier F, Ben Ghezala H, Razazi K, Mekontso-Dessap A, Brun-Buisson C. Risk factors for and prediction by caregivers of extubation failure in ICU patients: a prospective study. Crit Care Med. 2015;43(3):613-20.
46. Thille AW, Boissier F, Ben-Ghezala H, Razazi K, Mekontso-Dessap A, Brun-Buisson C, et al. Easily identified at-risk patients for extubation failure may benefit from noninvasive ventilation: a prospective before-after study. Crit Care. 2016;20(1):48.
47. Hernández G, Vaquero C, González P, Subira C, Frutos-Vivar F, Rialp G, et al. Effect of postextubation high-flow nasal cannula vs conventional oxygen therapy on reintubation in low-risk patients: a randomized clinical trial. JAMA. 2016;315(13):1354-61.
48. El-Solh AA, Aquilina A, Pineda L, Dhanvantri V, Grant B, Bouquin P. Noninvasive ventilation for prevention of post-extubation respiratory failure in obese patients. Eur Respir J. 2006;28(3):588-95.
49. Hernández G, Paredes I, Moran F, Buj M, Colinas L, Rodríguez ML, et al. Effect of postextubation noninvasive ventilation with active humidification vs high-flow nasal cannula on reintubation in patients at very high risk for extubation failure: a randomized trial. Intensive Care Med. 2022;48(12):1751-9.
50. Thille AW, Muller G, Gacouin A, Coudroy R, Decavèle M, Sonneville R, et al.; HIGH-WEAN Study Group and the REVA Research Network. Effect of postextubation high-flow nasal oxygen with noninvasive ventilation vs high-flow nasal oxygen alone on reintubation among patients at high risk of extubation failure: a randomized clinical trial. JAMA. 2019;322(15):1465-75.

51. Hernández G, Vaquero C, Ortiz R, Colinas L, de Pablo R, Segovia L, et al. Benefit with preventive noninvasive ventilation in subgroups of patients at high-risk for reintubation: a post hoc analysis. J Intensive Care. 2022;10(1):43.
52. Ferrer M, Sellarés J, Valencia M, Carrillo A, Gonzalez G, Badia JR, et al. Non-invasive ventilation after extubation in hypercapnic patients with chronic respiratory disorders: randomised controlled trial. Lancet. 2009;374(9695):1082-8.
53. Rochwerg B, Brochard L, Elliott MW, Hess D, Hill NS, Nava S, et al. Official ERS/ATS clinical practice guidelines: noninvasive ventilation for acute respiratory failure. Eur Respir J. 2017;50(2):1602426.
54. Keenan SP, Powers C, McCormack DG, Block G. Noninvasive positive-pressure ventilation for postextubation respiratory distress: a randomized controlled trial. JAMA. 2002;287(24):3238-44.
55. Esteban A, Frutos-Vivar F, Ferguson ND, Arabi Y, Apezteguía C, González M, et al. Noninvasive positive-pressure ventilation for respiratory failure after extubation. N Engl J Med. 2004;350(24):2452-60.
56. Lin C, Yu H, Fan H, Li Z. The efficacy of noninvasive ventilation in managing postextubation respiratory failure: a meta-analysis. Heart Lung. 2014;43(2):99-104.
57. Béduneau G, Pham T, Schortgen F, Piquilloud L, Zogheib E, Jonas M, et al.; WIND (Weaning according to a New Definition) Study Group and the REVA (Réseau Européen de Recherche en Ventilation Artificielle) Network. Epidemiology of Weaning Outcome according to a New Definition. The WIND Study. Am J Respir Crit Care Med. 2017;195(6):772-83.
58. Jaber S, Lescot T, Futier E, Paugam-Burtz C, Seguin P, Ferrandiere M, et al.; NIVAS Study Group. Effect of noninvasive ventilation on tracheal reintubation among patients with hypoxemic respiratory failure following abdominal surgery: a randomized clinical trial. JAMA. 2016;315(13):1345-53.
59. Palakshappa JA, Russell GB, Gibbs KW, Kloefkorn C, Hayden D, Moss M, et al.; NHLBI PETAL Network. Association of early sedation level with patient outcomes in moderate-to-severe acute respiratory distress syndrome: propensity-score matched analysis. J Crit Care. 2022;71:154118.
60. Huang DT, Angus DC, Moss M, Thompson BT, Ferguson ND, Ginde A, et al.; Reevaluation of Systemic Early Neuromuscular Blockade Protocol Committee and the National Institutes of Health National Heart, Lung, and Blood Institute Prevention and Early Treatment of Acute Lung Injury Network Investigators. Design and rationale of the reevaluation of systemic early neuromuscular blockade trial for acute respiratory distress syndrome. Ann Am Thorac Soc. 2017;14(1):124-33.
61. Ho KM, Finn J, Knuiman M, Webb SA. Combining multiple comorbidities with Acute Physiology Score to predict hospital mortality of critically ill patients: a linked data cohort study. Anaesthesia. 2007;62(11):1095-100.
62. Ho KM, Knuiman M, Finn J, Webb SA. Estimating long-term survival of critically ill patients: the PREDICT model. PLoS One. 2008;3(9):e3226.
63. Esper AM, Martin GS. The impact of comorbid [corrected] conditions on critical illness. Crit Care Med. 2011;39(12):2728-35.
